# Early Stage Relapsing Polychondritis Diagnosed by Nasal Septum Biopsy

**DOI:** 10.1155/2015/307868

**Published:** 2015-12-30

**Authors:** Takaaki Kobayashi, Sandra Moody, Masafumi Komori, Akira Jibatake, Makito Yaegashi

**Affiliations:** ^1^Department of Internal Medicine, Mount Sinai Beth Israel, New York, NY, USA; ^2^Department of Post-Graduate Education, Kameda Medical Center, Japan; ^3^Division of Geriatrics, University of California, San Francisco, CA, USA; ^4^Department of General Medicine, Kameda Medical Center, Japan; ^5^Department of Rheumatology and Allergy, Kameda Medical Center, Japan

## Abstract

Relapsing polychondritis is a rare inflammation of cartilaginous tissues, the diagnosis of which is usually delayed by a mean period of 2.9 years from symptom onset. We present the case of a 36-year-old man with nasal pain and fever. Physical examination of the nose was grossly unremarkable, but there was significant tenderness of the nasal bridge. Acute sinusitis was initially diagnosed due to thickened left frontal sinus mucosa on computed tomography (CT); however, there was no improvement after antibiotic intake. Repeat CT showed edematous inflammation of the nasal septum; biopsy of this site demonstrated erosion and infiltration of lymphocytes, plasma cells, eosinophils, and neutrophils in the hyaline cartilage. Relapsing polychondritis was confirmed by the modified McAdam's criteria and can be diagnosed at an early stage by nasal septum biopsy; it should be considered as a differential diagnosis in patients presenting with nasal symptoms alone or persistent sinus symptoms.

## 1. Introduction

Relapsing polychondritis (RP) is an immune-mediated condition involving cartilaginous structures and other tissues throughout the body. RP is a rare but severe systemic disease often misdiagnosed before the appearance of specific symptoms such as auricular inflammation or saddle nose deformity. No specific test is available; therefore, RP is clinically diagnosed. The mean delay in diagnosis is 2.9 years from symptom onset [1]. RP is associated with other autoimmune diseases such as ulcerative colitis (UC), even years after diagnosis [2].

## 2. Case

A 36-year-old man presented with the gradual onset of nasal pain and fever. One month before admission, he noticed an abnormal sensation around his nasal bridge, followed by progression to worsening pain. A few weeks before admission, he was prescribed oral faropenem for fever by his primary care physician, but his symptoms did not improve; this prompted him to consult our outpatient internal medicine clinic for further evaluation and treatment.

His past medical history was significant for small intestinal obstruction requiring surgery during childhood, the details of which were not available. He was in moderate distress due to the nasal pain. Physical examination revealed blood pressure of 112/60 mmHg; pulse rate of 64 beats/min; respiratory rate of 14 breaths/min; body temperature of 36.4°C; oxygen saturation of 98% in room air; and nasal bridge tenderness. Remarkable laboratory findings included white blood cell count, 15,500/*μ*L; hemoglobin, 11.7 g/dL; ALT, 53 U/L; CRP, 16.4 mg/dL; and ESR, 70 mm/h. Computed tomography (CT) revealed a thickened left frontal sinus mucosa.

**Figure 1:**
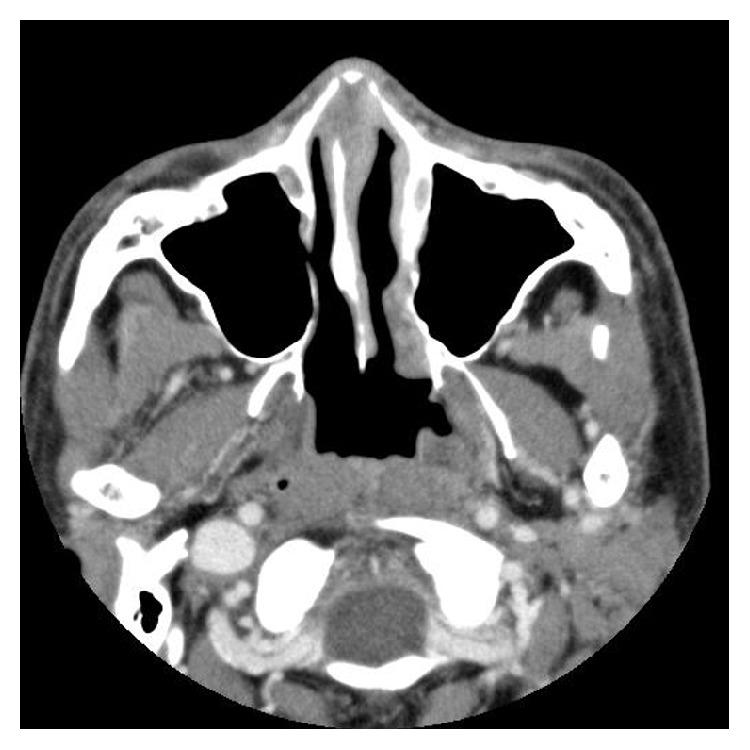
CT scan of a 36-year-old man with RP. The nasal septum is edematous and inflamed. CT, computed tomography; RP, relapsing polychondritis.

**Figure 2:**
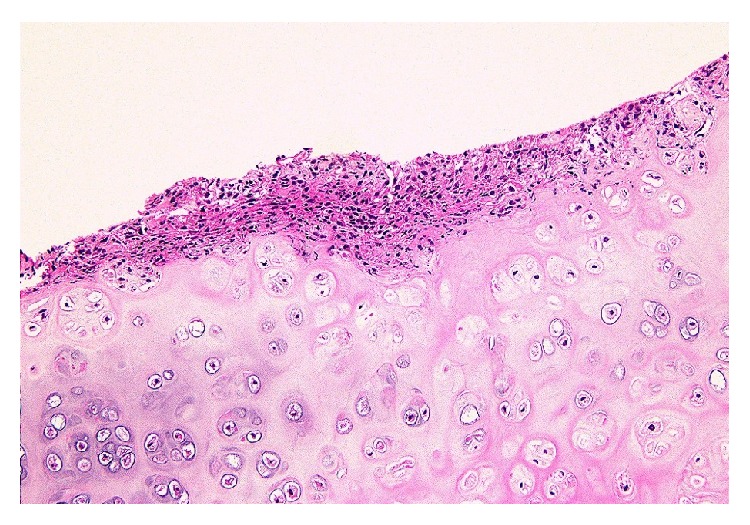
Photomicrograph of the nasal septum biopsy in a 36-year-old man with RP. The hyaline cartilage is eroded and infiltrated by lymphocytes, plasma cells, eosinophils, and neutrophils. (Hematoxylin and eosin stain, ×100). RP, relapsing polychondritis.

A tentative diagnosis of acute sinusitis was made based on the nasal bridge tenderness and CT findings. Oral amoxicillin/clavulanic acid was prescribed for 10 days but without any improvement. The nose pain became worse, and the patient was admitted for further evaluation. After admission, generalized joint pain and severe diarrhea developed. Repeat sinus CT revealed resolving mucosal thickening, but the nasal septum and cartilage were noted to be edematous and inflamed (Figure 1). Contrast magnetic resonance imaging confirmed the same findings. All serological tests, including those for rheumatoid factor and anti-neutrophil cytoplasmic antibodies (ANCA), were negative. Due to a suspicion of RP, the patient underwent nasal septum biopsy, which showed erosion and infiltration of lymphocytes, plasma cells, eosinophils, and neutrophils in the hyaline cartilage (Figure 2). The symptoms plus the biopsy findings fulfilled the modified McAdam's criteria for RP. Chest CT was negative for laryngotracheobronchial wall thickening, luminal narrowing, and cartilaginous calcification. Pulmonary function tests showed an obstructive pattern of the upper airways during inspiration. Retinal examination was unremarkable. Echocardiography and electrocardiography were also within normal limits.

**Figure 3:**
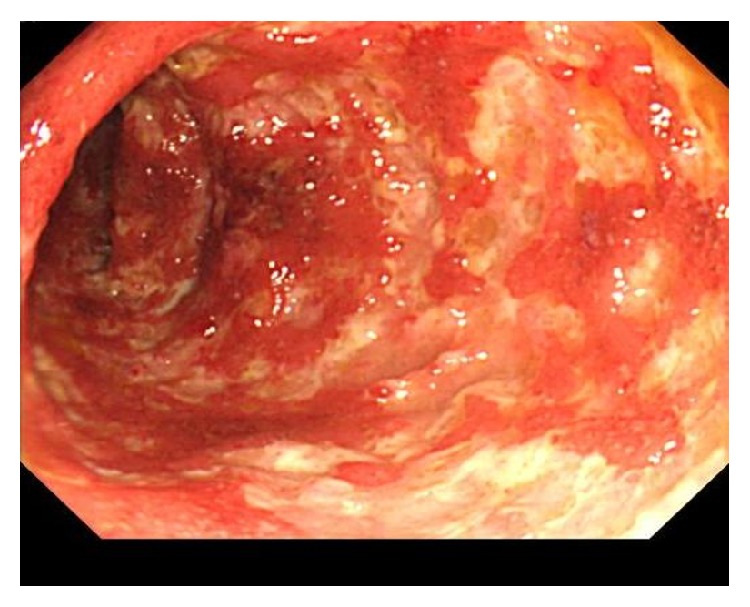
Colonoscopy findings in a 36-year-old man with RP who presented with bloody diarrhea five years after the initial diagnosis of RP. Multiple circular and longitudinal ulcers and erosions are seen in the descending colon. RP, relapsing polychondritis.

**Figure 4:**
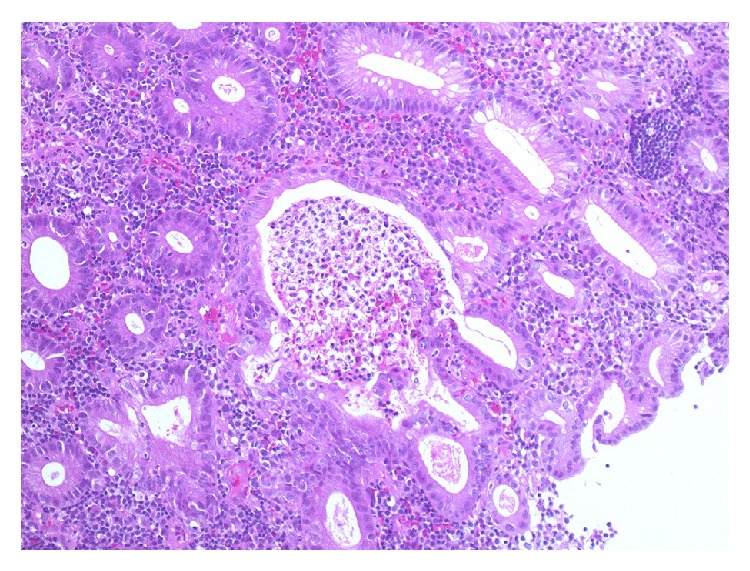
Photomicrograph of the colonic mucosal biopsy in a 36-year-old man with RP. There are crypt abscesses with infiltration of neutrophils and eosinophils. (Hematoxylin and eosin stain, ×100). RP, relapsing polychondritis.

Oral prednisone (30 mg daily) led to the gradual resolution of all symptoms, including polyarthralgia and diarrhea. The patient was stable on a maintenance dose of prednisone (15 mg daily) and methotrexate (16 mg weekly) until severe bloody diarrhea developed 5 years later. Colonoscopy demonstrated multiple ulcers with severe inflammation in the descending colon (Figure 3); biopsy showed crypt abscesses and the infiltration of inflammatory cells, including neutrophils and eosinophils (Figure 4); UC was diagnosed. Oral 5-aminosalicylic acid (5-ASA; 3600 mg daily) was initiated, and his prednisone dose was increased (60 mg daily). The patient's symptoms gradually resolved, and as of his last follow-up in June 2015, he remained stable on daily doses of prednisone (15 mg), 5-ASA (3600 mg), and azathioprine (75 mg).

## 3. Discussion

RP is a rare disease with an unknown etiology. In 1923, RP was first recognized by Jacksch–Wartenorst, who termed this disorder “polychodropathia.” In 1960, Pearson et al. renamed it as RP [3]. The diagnosis of RP is very difficult because of nonspecific symptoms, especially in early stages. Trentham and Le reported that the mean delay from symptom onset-to-diagnosis was 2.9 years, and one-third of patients went to more than five physicians before successful diagnosis [1]. In our case, we successfully diagnosed RP within 2 months of symptom onset, by performing a nasal septum biopsy to differentiate between Wegener's granulomatosis and RP. RP can mimic acute sinusitis, as in our patient, and should be considered in the differential diagnosis of persistent sinus symptoms such as fever and paranasal sinus tenderness.

There is no specific test for RP, and it is usually diagnosed by a combination of clinical and biopsy findings. In 1976, McAdam et al. established the diagnostic criteria that required the presence of more than three of the following six symptoms: auricular chondritis, polyarthritis, nasal chondritis, ocular inflammation, respiratory tract chondritis, and audiovestibular damage [2]. Our patient initially had only nasal symptoms. Although peripheral joint pain developed after admission, it did not meet the original McAdam's criteria. However, he fulfilled the modified McAdam's criteria for RP [4, 5], which required more than one of six symptoms plus histological confirmation or chondritis at two or more separate anatomic locations that is responsive to corticosteroids and/or dapsone. The initial change in histology involves the loss of basophilia in the cartilage matrix, corresponding to the loss of matrix proteoglycans. Additionally, a reduced number of chondrocytes and the infiltration of lymphocytes, neutrophils, and plasma cells in the cartilage–soft tissue interface are seen in areas of cartilage destruction [1]. The biopsy findings described in our case confirmed a diagnosis of RP. Therefore, we believe that the biopsy of affected regions is very helpful in obtaining an early diagnosis of RP.

Unilateral external ear inflammation, the most common presenting feature of RP, is seen in approximately 40% of patients and eventually appears in nearly 90% of patients [6]. Our patient only had nasal symptoms; there were no auricular or periauricular symptoms. Nasal chondritis is present at the time of diagnosis in 24% of patients and is seen at some stage of the disease in 53% of patients [7]. Saddle nose deformity is a typical RP finding, but our patient's nose was grossly unremarkable. Mucosal thickening on sinus CT raised the suspicion of Wegener's granulomatosis, which has been reported to occur in association with RP [8]. However, Wegener's granulomatosis usually has positive serology for ANCA and granulomas on biopsy specimens, findings that were not present in our patient.

Laryngotracheal disease occurs in more than half of patients and laryngotracheal stricture in nearly one quarter. Respiratory tract involvement accounts for almost 50% of deaths from RP [2]. Our patient did not show any abnormality between the inspiratory and expiratory phases on chest CT, but the flow–volume curve on spirometry revealed a variable extrathoracic upper airway obstructive pattern. This patient may have some degree of damage of the extrathoracic tracheal cartilages; therefore, regular pulmonary examinations are needed, and careful attention should be given to complaints of hoarseness, cough, dyspnea, choking sensations, wheezing, or stridor.

Other diseases associated with RP, including rheumatic disease, thyroid disease, and UC, have been reported; in fact, 25%–35% of patients with RP were found to have some associated autoimmune disease [2]. Five years after the diagnosis of RP, our patient was diagnosed with UC. Similar cases of RP patients developing UC, or vice versa, have been reported [9]. Coexistence of other autoimmune diseases, including UC, should be suspected if new symptoms such as severe diarrhea or bloody stools develop.

In conclusion, we encountered a case of RP that was diagnosed early by nasal septum biopsy. RP should be considered in patients who present with nasal symptoms alone or with persistent sinus symptoms. Coexistence of other autoimmune diseases should always be suspected if a patient with RP develops new symptoms.
